# Salinity signaling networks in wheat: crosstalk among Ca^2^⁺, ROS, phytohormones, and metabolic signals in salt adaptation

**DOI:** 10.1080/15592324.2026.2700909

**Published:** 2026-07-09

**Authors:** Hakim Zamir, Daud Ali Shah, Fawad Rauf, Hussam Ahmad, Shahrukh Khan, Usman Zulfiqar, Mohammed S. Alotaibi, Olimaxon Ergasheva, Dilnoza Sotiboldiyeva, Mayank Anand Gururani, Shakal Khan Korai

**Affiliations:** a Joint International Research Laboratory of Agriculture and Agri-Product Safety, The Ministry of Education of China, Institutes of Agricultural Science and Technology Development, Yangzhou University, Yangzhou, Jiangsu, People's Republic of China; b Jiangsu Key Laboratory of Crop Genetics and Physiology, Jiangsu Key Laboratory of Crop Genomics and Molecular Breeding, Yangzhou University, Yangzhou, People's Republic of China; c College of Bioscience and Biotechnology, Yangzhou University, Yangzhou, People's Republic of China; d College of Animal Science and Technology, Yangzhou University, Yangzhou, People's Republic of China; e Department of Agronomy, Faculty of Agriculture and Environment, The Islamia University of Bahawalpur, Bahawalpur, Pakistan; f Department of Biology, Nakhchivan State University, Nakhchivan, Azerbaijan; g Department of Biology, Turabah University College, Taif University, Taif, Saudi Arabia; h Department of Soil Science, National University of Uzbekistan named after Mirzo Ulugbek, Tashkent City, Uzbekistan; i Department of Botany and Genetics, National University of Uzbekistan, Tashkent, Uzbekistan; j Department of Biology, College of Science, United Arab Emirates University, Al Ain, United Arab Emirates

**Keywords:** Wheat, salinity stress, Ca^2+^ signaling, reactive oxygen species (ROS), phytohormones, ion homeostasis, signaling crosstalk

## Abstract

Soil salinity limits wheat productivity by disrupting water uptake, Na⁺/K⁺ homeostasis, photosynthesis, reproductive development, and grain filling. Although wheat salinity tolerance is often discussed in terms of individual traits such as Na⁺ exclusion, antioxidant defense, osmolyte accumulation, or abscisic acid signaling, these responses operate as interconnected signaling networks. This review reframes wheat salinity adaptation as a crosstalk-driven process linking early root perception with whole-plant acclimation and yield-related outcomes. At the root-soil interface, salinity rapidly lowers external water potential, alters membrane potential, disturbs ion fluxes, and induces early Ca^2^⁺, reactive oxygen species (ROS), pH, nitric oxide, electrical, and phosphorylation signals. Ca^2^⁺ sensors and decoders, including CaM/CMLs, CDPKs, and CBL-CIPK modules, connect these early signals with ROS regulation, ion-transporter activity, kinase cascades, and transcriptional reprogramming. ABA integrates osmotic stress with stomatal closure, hydraulic adjustment, compatible-solute accumulation, and water-use regulation, whereas additional hormonal and metabolic signals shape root architecture, growth restraint, senescence, source-sink balance, and reproductive protection. Wheat-specific evidence strongly supports the importance of *HKT1;5*-mediated Na⁺ retrieval, SOS-like ion regulation, K⁺ retention, antioxidant capacity, ABA-associated water regulation, osmotic adjustment, and genotype-dependent transcriptional responses. However, several important signaling models, including precise Ca^2^⁺ signatures, real-time Ca^2^⁺-ROS feedback dynamics, guard-cell ABA-ROS-Ca^2^⁺ signaling, systemic Ca^2^⁺/ROS waves, and salinity-specific sugar-redox-hormone control of grain filling, remain incompletely validated in wheat. By distinguishing wheat-supported mechanisms from conserved model-plant frameworks, this review identifies key signaling hubs and physiological trade-offs that may guide breeding, genome editing, priming, and agronomic strategies for improving wheat performance under saline environments.

## Graphical abstract

**Figure:**
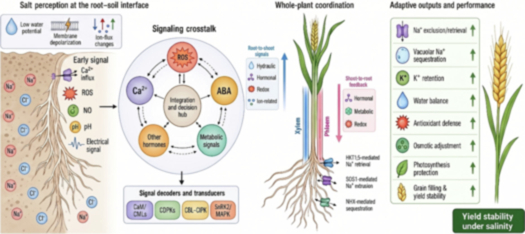

## 1. Introduction

Wheat is a major staple crop and a central contributor to global food and nutritional security, but its productivity is increasingly threatened by soil salinity.^1^ ^,^ ^2^ Salinity is particularly severe in irrigated, arid, semi-arid, and coastal production systems, where poor-quality irrigation water, high evapotranspiration, and climate-driven land degradation accelerate salt accumulation.^3^ ^,^ ^4^ The global scale of this constraint is substantial: more than 1,381 million hectares, approximately 10.7% of global land, are affected by salinity or sodicity.^5^ In wheat, salinity reduces performance across the life cycle, including germination, seedling establishment, root elongation, tillering, photosynthetic capacity, reproductive development, grain filling, and final yield.^6^ ^,^ ^7^

The physiological impact of salinity is complex because NaCl imposes osmotic, ionic, oxidative, hormonal, metabolic, and developmental constraints simultaneously.^8^ ^,^ ^9^ The earliest effect is osmotic: elevated salt in the rhizosphere lowers external water potential, restricts root water uptake, and rapidly suppresses cell expansion.^10^ With continued exposure, Na⁺ and Cl^−^ accumulate in plant tissues and cause ionic stress, disrupting K⁺ nutrition, membrane stability, enzyme activity, photosynthesis, and metabolic homeostasis.^11^ The classical two-phase model distinguishes a rapid osmotic phase from a slower ionic phase that accelerates tissue injury and senescence.^12^ In wheat, however, these phases overlap across tissues, developmental stages, and genotypes, requiring a network-based rather than pathway-by-pathway interpretation.^13^

Calcium, reactive oxygen species, phytohormones, metabolites, and ion transport systems are central to this coordination.^14^ Salt exposure rapidly activates Ca^2^⁺ influx, ROS production, cytosolic pH changes, nitric oxide signaling, electrical responses, and phosphorylation cascades.^15^ Ca^2^⁺ signatures are decoded by calmodulins, calmodulin-like proteins, calcium-dependent protein kinases, and CBL-CIPK modules, which regulate ion transport, antioxidant defense, hormone signaling, and transcriptional reprogramming.^16^ ^,^ ^17^ ROS function as signaling molecules that promote acclimation, but excessive ROS cause oxidative injury to lipids, proteins, nucleic acids, and photosynthetic structures.^18^ In wheat, salt-responsive Ca^2^⁺-linked kinases and redox-related genes, including *TaCDPK27* and *RBOH*-associated responses, support the relevance of Ca^2^⁺-ROS signaling, although direct real-time characterization of Ca^2^⁺-ROS dynamics remains limited.^19^

Hormonal and metabolic signals further shape wheat adaptation to salinity. Abscisic acid links osmotic stress with stomatal closure, hydraulic adjustment, osmolyte accumulation, and stress-responsive gene expression through the conserved PYR/PYL-PP2C-SnRK2 signaling module.^20^ Other hormones, including auxin, cytokinins, ethylene, gibberellins, brassinosteroids, jasmonates, and salicylic acid, regulate root architecture, shoot growth, senescence, defense activation, and reproductive development.^21^ Metabolites such as proline, soluble sugars, glycine betaine, GABA, phenolics, and flavonoids contribute to osmotic adjustment, redox buffering, carbon-nitrogen balance, and growth regulation.^22^

This network perspective is essential because the same signal may produce different outcomes depending on timing, intensity, tissue identity, genotype, and developmental stage.^23^ Controlled ROS production can activate acclimation, whereas excessive ROS promotes cellular damage.^24^ ^,^ ^25^ ABA-induced stomatal closure conserves water but may restrict CO₂ assimilation and reduce carbon supply for growth, ion transport, and grain filling.^26^ Na⁺ exclusion protects photosynthetic tissues, but active ion transport requires ATP, reducing power, and sustained source activity.^27^ Wheat salt tolerance therefore depends on the balance among Na⁺/K⁺ homeostasis, redox stability, osmotic adjustment, stomatal regulation, photosynthetic maintenance, source-sink coordination, and reproductive protection.^1^

A major gap in the current literature is that wheat salinity responses are often discussed as separate mechanisms, such as ion transport, antioxidant defense, ABA signaling, osmolyte accumulation, transcriptional regulation, or breeding targets. This separation does not fully explain how wheat coordinates local root perception with systemic shoot responses, developmental plasticity, reproductive-stage resilience, and yield stability. Wheat also presents crop-specific challenges because its hexaploid genome contains A, B, and D subgenomes, and many signaling and transporter genes occur as homoeologous copies with potential differences in expression, dosage, tissue specificity, or function.^16^ Thus, mechanisms inferred from *Arabidopsis,* rice, or barley must be evaluated carefully in wheat.

This review examines wheat salinity adaptation as an integrated signaling process. It synthesizes how Ca^2^⁺, ROS, phytohormones, metabolites, and ion transport systems interact to regulate early salt perception, signal amplification, Na⁺/K⁺ homeostasis, ABA-mediated water balance, growth plasticity, photosynthetic protection, source-sink regulation, developmental coordination, and grain filling. By distinguishing wheat-supported mechanisms from model-derived frameworks, this review provides a signaling-network perspective for improving wheat performance under saline environments.

## 2. Salt perception and early signal initiation in wheat

**Figure 1.**
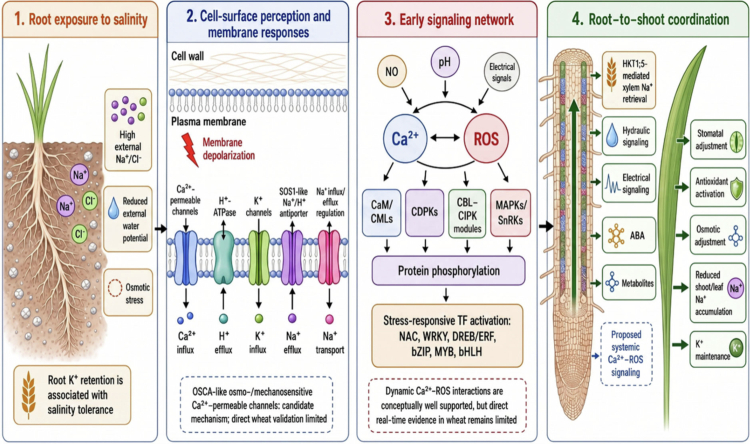
Early salinity perception and signal initiation in wheat roots. High external Na⁺ and Cl^−^ reduce external water potential, impose osmotic stress, disturb membrane potential, and alter ion fluxes at the wheat root surface. The cell wall-plasma membrane interface activates Ca^2^⁺ influx, H⁺-ATPase activity, K⁺ transport, *SOS1*-like Na⁺/H⁺ antiport, and Na⁺ influx/efflux regulation. These early responses generate Ca^2^⁺, ROS, NO, pH, electrical, and phosphorylation signals that are decoded by CaM/CMLs, CDPKs, CBL-CIPK modules, MAPKs/SnRKs, and stress-responsive transcription factors. These locally initiated signals activate ion regulation, antioxidant responses, hormonal signaling, and transcriptional reprogramming. Dashed elements indicate proposed or less directly validated mechanisms in wheat.

Salinity stress in wheat begins at the root-soil interface, where external NaCl rapidly reduces water potential, alters ion gradients, depolarizes the plasma membrane, and activates early signaling events. Within seconds to minutes, roots may show osmotic shock, Ca^2^⁺ influx, ROS production, nitric oxide generation, cytosolic pH shifts, electrical signaling, and protein phosphorylation; wheat-specific studies support the involvement of salt-responsive CDPKs, *TaCDPK27*, TaNOX-related ROS production, and early root transcriptomic reprogramming in these responses.^18^ ^,^ ^28^ ^,^ ^29^ These early responses link external salt exposure with downstream regulation of Na⁺ exclusion, K⁺ retention, osmotic adjustment, stomatal behavior, antioxidant defense, and growth modulation. In wheat, this early phase is especially important because root and xylem-based ion control directly influence shoot Na⁺ accumulation and yield performance under saline conditions. For example, *HKT1;5*-related Na⁺ exclusion loci reduce Na⁺ transport to leaves, and recent work identified *TaSPL6-D* as a regulator of *TaHKT1;5-D*, linking HKT-mediated Na⁺ regulation with salinity tolerance and yield-related traits in bread wheat.^27^ ^,^ ^30^ Thus, salt perception in wheat is best viewed as an integrated signaling process involving the cell wall, plasma membrane, ion channels, redox enzymes, Ca^2^⁺ sensors, protein kinases, and mobile root-shoot signals as summarized in Figure 1.^16^ ^,^ ^18^ ^,^ ^28^ ^,^ ^29^

### 2.1. Root-based perception of salinity stress

Roots are the first organs exposed to saline soil solution and therefore represent the primary site of salt perception in wheat. Increased external NaCl causes a sudden drop in soil water potential, which results in rapid osmotic stress before the accumulation of toxic concentrations of Na^+^ and Cl^−^ in shoots.^10^ This is followed by a slower ionic phase involving Na⁺/Cl^−^ accumulation, nutrient imbalance, oxidative stress, metabolic disruption, and leaf senescence. The two-phase model remains useful because early growth inhibition is largely associated with osmotic stress, whereas later tissue injury is mainly linked to ion toxicity.^31^ At the cellular level, high external Na⁺ alters electrochemical gradients, competes with K⁺ uptake, and induces membrane depolarization, thereby affecting ion-channel activity, Na⁺ influx, K⁺ retention, and cytosolic ion balance.^32^ These early root-exposure events correspond to the first stage of the proposed model, in which high external Na⁺/Cl^−^, reduced external water potential, and osmotic stress initiate membrane and signaling responses (Figure 1). Wheat-specific physiological evidence shows that root K⁺ retention is associated with salt tolerance, indicating that tolerance depends on maintaining a favorable K⁺/Na⁺ balance rather than simply minimizing Na⁺ accumulation.^33^ ^,^ ^34^

Genetic evidence further supports the importance of early root and vascular ion control. The *Nax2* locus in durum wheat and the *Kna1* locus in bread wheat are strongly associated with *HKT1;5*-type Na⁺ transporters, which contribute to xylem Na⁺ retrieval and reduce Na⁺ delivery to leaves.^35^ In durum wheat, the ancestral transporter *TmHKT1;5-A*, located at the *Nax2* locus, encodes a Na⁺-selective transporter expressed around root xylem vessels; its introgression reduced leaf Na⁺ accumulation and increased grain yield by approximately 25% under saline field conditions.^27^ More recently, *TaSPL6-D* was identified as a transcriptional repressor of *TaHKT1;5-D* in bread wheat; allelic variation that releases this repression enhances salinity tolerance while preserving yield-related traits, further supporting *HKT1;5*-mediated xylem Na⁺ control as a major wheat improvement target.^30^ Thus, early root and vascular ion regulation is not only a cellular salt-tolerance mechanism but also a field-relevant determinant of shoot ion homeostasis and yield stability.

### 2.2. Plasma membrane and cell wall as signaling platforms

The plasma membrane and cell wall form the first cellular interface where salinity-induced changes in ion gradients, membrane potential, membrane tension, and wall mechanics are converted into early signals. In wheat roots, Na⁺ efflux from epidermal cells depends on a *SOS1*-like plasma membrane Na⁺/H⁺ exchange system energized by H⁺-ATPase activity, with stronger activity reported in the salt-tolerant cultivar Kharchia 65 than in more sensitive genotypes.^36^ Ion channels and transporters at this interface regulate Na⁺, K⁺, Ca^2^⁺, H⁺, and Cl^−^ fluxes, thereby linking ionic balance with cytosolic Ca^2^⁺, pH, and electrical changes. Recent cereal evidence further shows that genotypic variation in root K⁺ and Ca^2^⁺ transporter sensitivity to H₂O₂ is associated with differential salt tolerance in wheat and barley, supporting a direct connection between ROS perception, cation-channel behavior, and root ion homeostasis.^37^ Thus, H⁺-ATPases, Na⁺/H⁺ antiporters, K⁺ channels, Ca^2^⁺-permeable channels, and HKT-type transporters are key early components linking salt exposure with cellular responses.

Osmotic shock also changes turgor and membrane tension, potentially activating mechanosensitive or osmosensitive Ca^2^⁺-permeable channels. In *Arabidopsis*, *OSCA1* provides direct molecular evidence for osmotic-to-Ca^2^⁺ coupling, as it mediates osmotic-stress-induced Ca^2^⁺ increases.^38^ In wheat, 42 *TaOSCA* genes have been identified, and expression profiling suggests roles in development and stress responses; however, their direct function in wheat salt perception still requires validation.^39^ The cell wall is also an active part of early perception. *Arabidopsis FERONIA* links salt-induced cell-wall integrity changes with Ca^2^⁺ signaling, while recent cell-wall syntheses highlight pectin remodeling, wall stiffness, and wall-membrane adhesion as important salinity-response features.^40^ ^,^ ^41^ In wheat, this wall-based layer remains less characterized than transporter-mediated ion regulation, but it is relevant because root elongation, membrane stability, and cell expansion are among the earliest processes inhibited by salinity, and wheat root salt responses involve coordinated changes in ion transport, ROS signaling, and growth regulation.^16^ ^,^ ^29^

### 2.3. Early second-messenger initiation

Following membrane-level salt perception, wheat roots rapidly generate interacting second messengers that convert osmotic and ionic disturbances into intracellular signals. These early responses include cytosolic Ca^2^⁺ elevation, ROS production, nitric oxide generation, cytosolic pH shifts, electrical activity, and protein phosphorylation.^15^ ^,^ ^18^ ^,^ ^28^ ^,^ ^29^ Rather than functioning independently, these signals form an early signaling interface that links changes in external water potential, membrane potential, and ion fluxes with downstream transporter regulation, antioxidant activation, hormonal responses, and transcriptional reprogramming.

Ca^2^⁺ and ROS are central components of this initial response. Salt-induced Ca^2^⁺ elevations are perceived by CaM/CMLs, CDPKs, and CBL-CIPK modules, whereas early ROS production is primarily associated with plasma-membrane RBOH/NADPH oxidases.^17^ ^,^ ^18^ ^,^ ^42^ ^,^ ^43^ Wheat functional and transcriptomic studies support the involvement of Ca^2^⁺-linked kinases, RBOH-associated ROS production, redox regulation, and genotype-dependent signaling responses under salinity.^16^ ^,^ ^18^ ^,^ ^28^ ^,^ ^29^ However, the precise timing, cellular origin, and tissue specificity of these signals remain incompletely characterized in wheat.

### 2.4. Transition from local perception to systemic coordination

Although salinity is initially perceived by root epidermal and cortical cells, effective wheat adaptation requires rapid communication with vascular, leaf, and reproductive tissues. Locally generated Ca^2^⁺, ROS, nitric oxide, pH, electrical, and phosphorylation signals interact with hydraulic changes caused by reduced root water uptake, ABA-associated signaling, and mobile metabolites such as sugars, amino acids, organic acids, and peptides.^44-46^ Systemic Ca^2^⁺ and ROS signaling may further contribute to root-to-shoot communication through regenerative cell-to-cell propagation, although these mechanisms are better established in model plants than in wheat.^47^ ^,^ ^48^ Wheat-specific evidence for HKT1;5-mediated xylem Na⁺ retrieval and root K⁺ retention nevertheless confirms the importance of coordinating early root signaling with long-distance ion regulation.^27^ ^,^ ^49^

This transition involves both rapid information transfer and the subsequent physical redistribution of ions. Hydraulic, electrical, hormonal, and regenerative Ca^2^⁺-ROS signals may activate shoot acclimation before Na⁺ reaches damaging concentrations, whereas long-distance Na⁺ transport is controlled through tissue-specific loading, retrieval, exclusion, and compartmentalization mechanisms.

## 3. Ca^2^⁺-ROS signal encoding, feedback, and propagation

**Figure 2.**
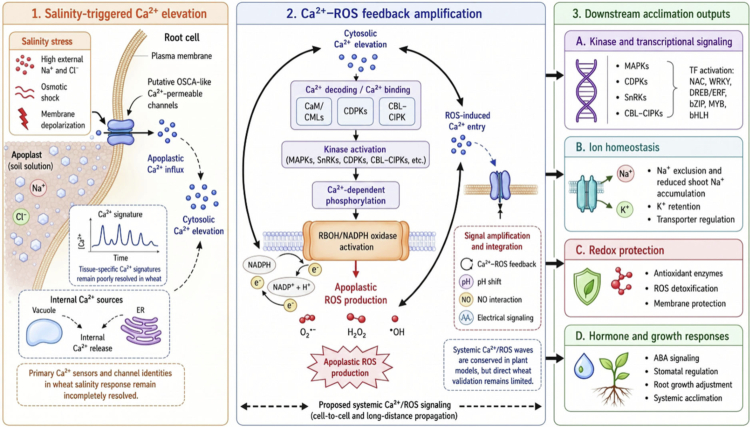
Ca^2^⁺-ROS feedback signaling in salt-stressed wheat. Salinity triggers Ca^2^⁺ elevation through Ca^2^⁺-permeable channels and possible internal Ca^2^⁺ release, while also inducing membrane depolarization and osmotic shock. Cytosolic Ca^2^⁺ is decoded by CaM/CMLs, CDPKs, and CBL-CIPK modules, leading to kinase activation, Ca^2^⁺-dependent phosphorylation, and RBOH/NADPH oxidase-mediated apoplastic ROS production. ROS, especially H₂O₂, can promote further Ca^2^⁺ entry, forming a reciprocal Ca^2^⁺-ROS feedback loop. This signaling network integrates pH shifts, nitric oxide, and electrical signals to regulate transcriptional responses, ion homeostasis, redox protection, ABA-related stomatal regulation, root growth adjustment, and systemic acclimation. Dashed elements indicate proposed or less directly validated mechanisms in wheat.

Following their initiation during root salt perception, Ca^2^⁺ and ROS signals are spatially and temporally encoded, decoded, and amplified to generate appropriate downstream responses. Ca^2^⁺ conveys information through changes in signal amplitude, duration, frequency, and cellular distribution, whereas ROS function as concentration-, location-, and time-dependent redox signals that can either promote acclimation or cause oxidative damage.^15^ ^,^ ^25^ ^,^ ^42^ Reciprocal Ca^2^⁺-ROS interactions connect early salt perception with kinase activation, ion-transport regulation, antioxidant defense, hormone signaling, transcriptional reprogramming, and systemic acclimation.^15^ ^,^ ^50^ Figure 2 summarizes Ca^2^⁺ signal encoding, reciprocal Ca^2^⁺-ROS feedback amplification, and the resulting downstream responses. Although wheat studies support the involvement of Ca^2^⁺ sensors, protein kinases, RBOH enzymes, antioxidant systems, and salt-responsive transcription factors, the real-time spatial dynamics of Ca^2^⁺-ROS signaling remain less well characterized in wheat than in *Arabidopsis* and other model plants.

### 3.1. Salt-induced Ca^2^⁺ signatures in wheat

Salt exposure rapidly increases cytosolic Ca^2^⁺, making Ca^2^⁺ one of the earliest signals in plant salinity responses.^51^ In plants, Ca^2^⁺ elevations differ in amplitude, duration, frequency, and cellular location, forming stimulus-specific “Ca^2^⁺ signatures” that are decoded by calmodulins, calmodulin-like proteins, calcium-dependent protein kinases, and CBL-CIPK modules.^52^ These decoders convert Ca^2^⁺ changes into phosphorylation events, transporter regulation, antioxidant activation, and stress-responsive gene expression.

In wheat, evidence for Ca^2^⁺ involvement comes from both physiological and molecular studies. Transcriptome analyzes across contrasting bread wheat genotypes indicate that calcium-binding proteins and transcription factors are differentially regulated during the early osmotic phase of salt stress, supporting genotype-dependent Ca^2^⁺-linked signaling in wheat. However, the precise tissue-specific Ca^2^⁺ signatures in wheat roots, leaves, guard cells, vascular tissues, and reproductive organs remain insufficiently resolved.^53^ ^,^ ^54^ Therefore, claims about wheat Ca^2^⁺ signatures should be framed as strongly supported by conserved plant signaling principles and wheat gene-expression/functional evidence, but not yet fully defined by live-cell imaging. Consistent with this evidence gap, Figure 2 depicts salinity-triggered cytosolic Ca^2^⁺ elevation and possible internal Ca^2^⁺ release while noting that primary Ca^2^⁺ sensor and channel identities in wheat salinity responses remain incompletely resolved.

### 3.2. ROS generation as an early salinity signal

ROS are generated rapidly after salt exposure and function as early redox signals during salinity adaptation.^55^ ^,^ ^56^ Apoplastic ROS are mainly produced by plasma membrane respiratory burst oxidase homologs, while chloroplasts, mitochondria, and peroxisomes become important ROS sources when salinity disrupts photosynthesis, respiration, photorespiration, and cellular redox balance.^57^ Hydrogen peroxide is particularly significant since it is more stable than superoxide and can enter cells through aquaporins as well as act locally and connect extracellular stress perception to intracellular responses.^56^

The roles of ROS are context, concentration and time dependent. Controlled ROS production activates antioxidant enzymes, stress-responsive genes, ion-transport regulation, and acclimation pathways, whereas excessive ROS damages lipids, proteins, nucleic acids, and photosynthetic components.^55^ Wheat evidence supports this dual role.^58^ Early root transcriptome analysis of synthetic hexaploid and common wheat showed that salt stress altered redox-related genes and that several *RBOH* genes were up-regulated over time in salt-tolerant synthetic wheat but not in common wheat lines, suggesting genotype-specific ROS signaling during early salt response. The same study found differential regulation of kinases, transporters, SOS-related genes, and transcription factors, indicating that ROS-related responses operate within a broader signaling network rather than as isolated oxidative damage responses.^16^ Accordingly, Figure 2 presents RBOH/NADPH oxidase-mediated apoplastic ROS production as a central amplification step linking Ca^2^⁺-dependent signaling with redox protection, ion homeostasis, and transcriptional regulation.

### 3.3. Ca^2^⁺-ROS feedback loops

Ca^2^⁺ and ROS reinforce each other through reciprocal feedback. Ca^2^⁺ can stimulate ROS production by activating RBOH proteins directly through EF-hand Ca^2^⁺-binding domains or indirectly through Ca^2^⁺-dependent phosphorylation.^18^ ^,^ ^59^ ^,^ ^60^ In turn, ROS can activate Ca^2^⁺-permeable channels, causing additional Ca^2^⁺ influx. This feedback creates a signal-amplification system in which Ca^2^⁺ promotes ROS generation and ROS strengthens Ca^2^⁺ signaling.^61^ In plants, *RBOH* activity is regulated by both Ca^2^⁺ binding and phosphorylation, allowing Ca^2^⁺ sensors, CDPKs, MAPKs, and other kinases to connect salinity perception with redox signaling.^18^ ^,^ ^60^ This reciprocal Ca^2^⁺-ROS amplification model shows cytosolic Ca^2^⁺ elevation, Ca^2^⁺ decoding, kinase activation, RBOH/NADPH oxidase activity, apoplastic ROS production, and ROS-linked Ca^2^⁺ entry form an integrated feedback module. In wheat, this feedback model is supported indirectly by functional and transcriptomic evidence.

The salt-responsive kinase *TaCDPK27* contributes to salt tolerance and helps limit excessive ROS accumulation and salt-induced cell death, linking Ca^2^⁺ decoding with redox homeostasis.^17^ Similarly, genotype-specific induction of *RBOH*, redox-related genes, kinases, and transcription factors in salt-tolerant synthetic wheat suggests that Ca^2^⁺- and ROS-associated pathways are activated early during salt acclimation.^54^ These Ca^2^⁺-ROS-linked processes can contribute to downstream acclimation outputs, including kinase and transcriptional signaling, ion homeostasis, antioxidant/redox protection, ABA signaling, stomatal regulation, root growth adjustment, and systemic acclimation. Nitric oxide, cytosolic pH shifts, electrical signals, ABA, and phosphorylation cascades probably further modulate this Ca^2^⁺-ROS network, but their causal relationships in wheat require more direct validation through mutants, overexpression lines, CRISPR/Cas editing, and homoeolog-specific analyzes.

### 3.4. Signal amplification and propagation

After local root salt perception, Ca^2^⁺ and ROS signals must be amplified and coordinated across tissues. In model plants such as *Arabidopsis*, localized salt stress can trigger systemic Ca^2^⁺ waves that move from root to shoot and activate stress-responsive gene expression in distant tissues.^47^ Studies show that systemic signaling depends on a ROS-assisted calcium-induced calcium-release mechanism involving *RBOHD*-dependent ROS production and *TPC1*-dependent Ca^2^⁺ release.^48^ This supports a model in which ROS diffusion, ROS-induced Ca^2^⁺ entry, Ca^2^⁺-induced ROS production, membrane depolarization, and vacuolar Ca^2^⁺ release cooperate to propagate stress information beyond the initially stressed root cells.^45^ ^,^ ^62^

For wheat, Ca^2^⁺-ROS wave propagation should be presented as a conserved and testable model rather than a fully demonstrated mechanism. Direct real-time imaging of systemic Ca^2^⁺-ROS dynamics under salt stress remains limited in wheat. Nevertheless, wheat physiological and molecular evidence supports the broader need for rapid root-to-shoot integration. Salt-responsive wheat transcriptomes show early changes in kinases, redox genes, RBOHs, SOS-related transporters, and transcription factors, while functional studies of Ca^2^⁺ decoders such as *TaCDPK27* link calcium signaling with antioxidant balance and salt tolerance.^16^ ^,^ ^17^ ^,^ ^29^ Such responses provide a mechanistic bridge between local root salt perception and systemic acclimation processes, including stomatal adjustment, antioxidant activation, osmotic regulation, Na⁺/K⁺ homeostasis, and growth control.

Overall, early Ca^2^⁺ and ROS signals in wheat salinity responses are generated through plasma membrane ion fluxes, apoplastic and organellar redox activity, and intracellular Ca^2^⁺ mobilization. These signals are then amplified through Ca^2^⁺ decoders, RBOH-mediated ROS production, kinase cascades, antioxidant regulation, and transcriptional reprogramming. The strongest wheat-specific evidence currently supports the involvement of calcium-binding proteins, CDPKs such as *TaCDPK27*, *RBOH*/redox-related genes, SOS-associated transporters, and stress-responsive transcription factors.^16^ ^,^ ^17^ ^,^ ^29^ ^,^ ^54^ By contrast, detailed Ca^2^⁺-ROS wave propagation remains better established in *Arabidopsis* and should be treated as a model requiring direct wheat validation.

## 4. Connection of Ca^2^⁺-ROS signals with ion transport and Na⁺/K⁺ homeostasis

A major function of early Ca^2^⁺-ROS signaling under salinity is to connect stress perception with ion-transport regulation. Salt tolerance in wheat is related to the ability to limit the accumulation of Na^+^ in the cytosol, ensure the availability of K^+^ to the roots, restrict xylem Na^+^ movement and prevent damage to photosynthetic and reproductive tissues by Na^+^ and/or Cl^−^ toxicity.^2^ ^,^ ^57^ Ca^2^⁺ signals are decoded by kinase-based modules that regulate transporters, whereas ROS influence membrane redox status, ion-channel activity, kinase signaling, antioxidant defense, and transporter expression. Ca^2^⁺ and ROS help determine whether transport responses favor Na⁺ exclusion, vacuolar sequestration, K⁺ retention, or ion leakage.^9^ ^,^ ^15^ ^,^ ^37^ ^,^ ^61^ The major signaling and homeostasis modules that link early Ca^2^⁺-ROS signaling with wheat salinity tolerance are summarized in Table 1. Wheat-specific evidence from *HKT1;5*-linked Na⁺ exclusion loci, *TaSOS1* homoeologs, *TaNHX* transporters, and root K⁺ retention shows that ion homeostasis is a central physiological and genetic component of salinity tolerance.^63-65^

**Table 1.** Key signaling and regulatory modules in wheat salinity responses. Summary of major wheat-supported signaling, transport, antioxidant, hormonal, metabolic, and transcriptional modules involved in salt-stress adaptation.

Module	Key components	Signaling role	Wheat evidence/status	References
Ca^2^⁺ signaling	CaM/CMLs, CDPKs, CBL-CIPKs	Decodes salt-induced Ca^2^⁺ signals and links them to phosphorylation, transcription, and ion regulation	Wheat evidence includes salt-responsive Ca^2^⁺-binding proteins, *TaCDPK27*, and *TaCIPK25*-linked salt responses	[17,54,66]
ROS production	RBOHs*/NOXs*	Generates apoplastic ROS for signal amplification and stress-response activation	Wheat transcriptomes report salt-responsive RBOHs; *TaNOX7*-mediated ROS production is functionally supported	[16,18]
Antioxidant defense	SOD, CAT, APX, GR, POD	Scavenges ROS and protects redox homeostasis under salinity	Tolerant wheat genotypes show stronger antioxidant activity, lower MDA, and better PSII/membrane protection	[58,67,68]
ABA signaling	ABA, PP2Cs, SnRK2s, ABA-responsive regulators	Coordinates osmotic signaling, transpiration, ion balance, and antioxidant responses	ABA improves wheat salt/alkali tolerance; *TaSnRK2.8* and *TaPP2C*-linked salt responses are reported	[69–72]
Na⁺ exclusion/retrieval	*HKT1;5*, *SOS1*	Limits shoot Na⁺ accumulation through xylem retrieval and Na⁺ extrusion	Strong wheat evidence for *TmHKT1;5-A*/*Nax2*, *TaHKT1;5-D*/*Kna1*, and *TaSOS1* homoeologs	[27,35,63,73,74]
Vacuolar sequestration	NHX, H⁺-ATPase, H⁺-PPase	Uses proton gradients to compartmentalize Na⁺/K⁺ and protect cytosolic ion balance	Wheat evidence includes *TaNHX1*/*TVP1*, *TaNHX2*, and TaNHX family expression under salinity	[64,75,76]
K⁺ homeostasis	K⁺ channels, K⁺ transporters	Maintains K⁺ uptake/retention and favorable K⁺/Na⁺ balance	Root K⁺ retention and higher K⁺/Na⁺ ratios are associated with wheat salt tolerance	[33,49,77]
Hormone crosstalk	Auxin, CK, ethylene, GA, BR, JA, SA	Balances growth, defense, root architecture, and stress recovery	Wheat evidence is strongest for BR/auxin-linked *TaGSK3* root plasticity and BR-supported root salt tolerance	[78–80]
Metabolic adjustment	Proline, sugars, amino acids/GABA, organic acids, phenolics/glutathione	Supports osmotic adjustment, energy balance, and redox buffering	Wheat metabolomic and transcriptome-metabolome studies show salt/salt-alkali metabolic remodeling	[81–84]
Transcriptional control	NAC, WRKY, DREB/ERF, bZIP, MYB, bHLH	Reprograms salt-responsive genes for signaling, transport, redox control, and growth adjustment	Wheat root, leaf, and comparative transcriptomes identify salt-responsive TF families	[14,16]

Note: Wheat evidence is strongest for ion homeostasis, antioxidant defense, ABA-linked physiology, metabolite remodeling, and transcriptomic regulation. Some upstream signaling components and crosstalk mechanisms remain partly inferred from model plants.

### 4.1. Ca^2^⁺ decoding under salinity

Salt-induced Ca^2^⁺ signals link external NaCl exposure with transporter regulation, antioxidant defense, and transcriptional reprogramming.^85^ Ca^2^⁺ itself does not execute most downstream responses; it is decoded by Ca^2^⁺-binding proteins, including calmodulins, calmodulin-like proteins, calcium-dependent protein kinases, and CBL-CIPK modules. These decoders translate transient Ca^2^⁺ changes into phosphorylation events, protein-protein interactions, transporter activation, and gene-expression changes.^54^ ^,^ ^86^ CaM/CMLs, CDPKs and CBL-CIPK modules are the core components of Ca^2+^-signaling that link Ca^2+^-signals with signal decoding, ion regulation and downstream stress response.

This decoding is particularly relevant to ion homeostasis, as various tissues have different transport output demands. Root epidermal and cortical cells must restrict excessive Na⁺ entry while maintaining K⁺ uptake; xylem parenchyma cells regulate Na⁺ loading and retrieval from the transpiration stream; leaf mesophyll cells require ionic and redox stability; and guard cells integrate ion fluxes with stomatal control. In wheat, functional evidence for Ca^2^⁺ decoding is emerging. The salt-responsive calcium-dependent protein kinase *TaCDPK27* is positively involved in salt tolerance and is correlated with enhanced ROS balance and less salt induced cell injury, thus connecting the Ca2^+^-signaling and the redox regulation with the stress protection.^17^ However, the direct phosphorylation targets of most wheat Ca^2^⁺ decoders remain unresolved, and identifying their transporter targets is an important step toward connecting early Ca^2^⁺ signals with Na⁺/K⁺ homeostasis.^85^ ^,^ ^86^

### 4.2. CBL-CIPK and SOS-like signaling in wheat

CBL-CIPK modules are a key molecular connection between Ca^2+^ signals and ion transport under salinity.^87^ In the classical Salt Overly Sensitive pathway, salt-induced Ca^2^⁺ signals are perceived by CBL proteins, which recruit and activate CIPK kinases. The CBL-CIPK module then regulates ion transport, especially through activation of the plasma membrane Na⁺/H⁺ antiporter *SOS1*, promoting Na⁺ extrusion from the cytosol.^88^ ^,^ ^89^ Initial studies in *Arabidopsis* revealed that *SOS1* is a plasma membrane Na^+^/H^+^-exchanger and that *SOS2/CIPK24* and *SOS3/CBL4* are activators of *SOS1* activity, thus defining the SOS pathway as a Ca^2+^-dependent Na^+^ homeostasis mechanism.^87^ ^,^ ^89^

In wheat, SOS-like signaling appears conserved but is complicated by hexaploidy and homoeolog-specific regulation. Wheat has multiple *SOS1* related homoeologs and recent work revealed that *TaSOS1-A*, *TaSOS1-B* and *TaSOS1-D* exhibit subgenome-biased expression and functional divergence under salt stress. Among these, *TaSOS1-D* showed a prominent contribution to salt tolerance in heterologous functional assays, indicating that wheat *SOS1* homoeologs are not functionally equivalent.^63^ This reinforces an important concept for wheat salinity biology: conserved SOS principles are beneficial, but need to be evaluated in a polyploid, tissue-specific and genotype-dependent context. Therefore, future work should identify which wheat CBL-CIPK combinations regulate *SOS1* activity, HKT-mediated xylem retrieval, NHX-mediated vacuolar sequestration, and K⁺ retention in specific tissues and developmental stages.

### 4.3. Regulation of Na⁺ exclusion, sequestration, and transport

Following early Ca^2^⁺-ROS signaling, wheat must restrict toxic Na⁺ accumulation in metabolically active tissues. This includes coordinated Na^+^ extrusion from the root cells, limitation of Na^+^ loading in the xylem, reabsorption of Na^+^ from the transpiration stream, and accumulation of Na^+^ in vacuoles. HKT-type transporters are especially important for long-distance Na⁺ regulation. In wheat, *HKT1;5*-like transporters are strongly associated with major Na⁺ exclusion loci, including *Nax2* in durum wheat and *Kna1* in bread wheat, which reduce shoot Na⁺ accumulation by regulating Na⁺ transport around xylem tissues.^35^ ^,^ ^73^

The agronomic importance of this mechanism has been demonstrated in durum wheat. The ancestral transporter gene *TmHKT1;5-A*, located at the *Nax2* locus, encodes a Na⁺-selective transporter localized around root xylem vessels. It withdraws Na⁺ from the transpiration stream, reduces Na⁺ delivery to leaves, and improves durum wheat grain yield by about 25% under saline field conditions in near-isogenic lines.^27^ ^,^ ^90^ Additional work on *Nax1* and *Nax2* showed that these ancestral Na⁺ exclusion loci reduce leaf Na⁺ accumulation, although their yield effects may differ depending on genetic background and salinity level.^90^ ^,^ ^91^ These studies provide strong wheat-specific evidence that xylem Na⁺ retrieval is a field-relevant component of salinity tolerance.

Na⁺ sequestration into vacuoles provides a second protective mechanism by reducing cytosolic Na⁺ toxicity while allowing compartmentalized Na⁺ to contribute to osmotic adjustment. NHX-type Na^+^/H^+^ antiporters are able to compartmentalize cations using proton gradients generated by H^+^-ATPases and H^+^-PPases. Genome-wide analysis of the wheat NHX family revealed several *NHX* genes that are potentially involved in salinity responses,^64^ and previous functional work suggests that *TaNHX1* and *TaNHX2* may contribute to salt tolerance through Na⁺/H⁺ exchange and vacuolar ion compartmentalization.^75^ ^,^ ^92^ Consistent with this evidence, Table 1 lists NHX transporters, H⁺-ATPases, and H⁺-PPases as key components of vacuolar sequestration, with cytosolic Na⁺ protection and osmotic adjustment as major outputs. But wheat NHX functions are still more directly to be validated in planta, particularly by the use of homoeolog-specific mutants and tissue-specific expression analyzes, compared with *HKT1;5*-mediated xylem Na^+^ retrieval.

### 4.4. Maintenance of K⁺ uptake and retention

Maintenance of K⁺ homeostasis is as important as Na⁺ exclusion during salinity stress. High external Na⁺ competes with K^+^ uptake, induces K^+^ efflux and decreases the cytosolic K^+^/Na^+^ ratio.^77^ K⁺ is essential for enzyme activation, protein synthesis, membrane potential, osmotic balance, stomatal movement and phloem transport, and loss of K⁺ may also affect growth when accumulation of Na⁺ is partly controlled.^93^ ^,^ ^94^ Therefore, salt tolerance depends on preserving a favorable K⁺/Na⁺ balance rather than simply reducing Na⁺ concentration.

Wheat-specific physiological evidence strongly supports this point. Under NaCl stress, a correlation between K^+^ flux and major physiological traits and yield related performance of wheat roots was observed, suggesting that NaCl-induced K^+^ flux could be used as a screening marker for salt tolerance.^49^ Regulation of K⁺ retention involves K⁺ channels, K⁺ transporters, H⁺ pumps, Ca^2^⁺ signaling, ROS levels, membrane potential and phosphorylation-dependent control.^65^ Moderate Ca^2^⁺ signaling can stabilize membranes and regulate ion channels, whereas excessive ROS and membrane depolarization may activate K⁺ efflux pathways and increase ion leakage.^37^ Therefore, efficient Ca^2+^-ROS regulation is crucial not only for Na^+^-restriction, but also for maintaining K^+^-uptake and retention.

### 4.5. ROS, ABA, and metabolic regulation of ion homeostasis

Ion transport under salinity is embedded within a broader regulatory network involving ROS, ABA, and metabolic status.^12^ ROS can directly modify transport by altering the redox state of membranes and indirectly modify transport by stimulating kinases, phosphatases, transcription factors and antioxidant systems.^51^ At controlled levels, ROS contribute to transporter signaling and acclimation; at excessive levels, they damage membranes, disrupt transporter function, and increase ion leakage.^37^ ROS production is related to RBOH/NADPH oxidase activity, Ca^2^⁺-ROS signaling and defense activation, and antioxidant defense is related to SOD, CAT, APX, GR, and POD, which limit oxidative damage and maintain redox balance. ABA links osmotic stress with stomatal closure, root-to-shoot signaling, transporter expression, and hydraulic regulation, thereby influencing the demand for ion transport and water conservation.^95^

The energetic cost of ion homeostasis is substantial. The extrusion of Na⁺ across the plasma membrane and sequestration into vacuoles depends on proton gradients generated by H^+^-ATPases and H^+^-PPases which require ATP, reducing power and carbon supply.^96^ Therefore, sugar availability is not only a metabolic resource but also a determinant of the plant’s capacity to sustain active Na⁺ transport, K⁺ uptake, and vacuolar compartmentalization.^97^ Salinity-induced stomatal closure or chloroplast damage can cause a reduction in photosynthesis, which can decrease carbohydrate supply and thus the energy available for ion homeostasis. In wheat, this links early Ca^2^⁺-ROS signaling with later source-sink balance, because roots require shoot-derived carbon to maintain Na⁺ exclusion, K⁺ retention, membrane repair, and antioxidant defense.^2^

Overall, early Ca^2^⁺-ROS signaling is translated into Na⁺/K⁺ homeostasis through Ca^2^⁺ decoders, CBL-CIPK/SOS-like modules, redox-sensitive transporter regulation, HKT-mediated xylem Na⁺ retrieval, NHX-dependent sequestration, K⁺ retention mechanisms, and energy-dependent proton gradients.^15^ ^,^ ^86^ Currently, the most compelling evidence for wheat is *HKT1;5*/Nax/*Kna1*-mediated Na⁺ exclusion, *TaSOS1* homoeolog diversification, root K⁺ retention and candidate *TaNHX*-mediated vacuolar sequestration.^2^ ^,^ ^64^ ^,^ ^75^ These mechanisms provide the physiological foundation for osmotic adjustment, photosynthetic maintenance, reproductive protection, and yield stability under saline conditions.

## 5. ABA uses Ca^2^⁺ and ROS to control water balance

**Figure 3.**
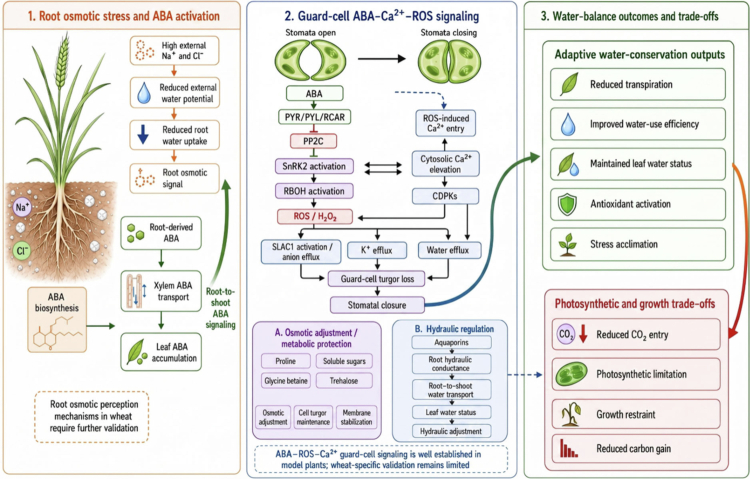
ABA-Ca^2^⁺-ROS regulation of water balance in salt-stressed wheat. Salinity reduces external water potential and root water uptake, triggering osmotic signaling, ABA biosynthesis, xylem ABA transport, and leaf ABA accumulation. In guard cells, ABA signaling through the PYR/PYL/RCAR-PP2C-SnRK2 pathway promotes *RBOH*-mediated ROS/H₂O₂ production, ROS-induced Ca^2^⁺ entry, cytosolic Ca^2^⁺ elevation, CDPK activation, anion and K⁺ efflux, guard-cell turgor loss, and stomatal closure. ABA also supports osmotic adjustment through compatible solutes and contributes to hydraulic regulation through aquaporins, root hydraulic conductance, root-to-shoot water transport, and leaf water status. These responses improve water conservation, water-use efficiency, antioxidant activation, and stress acclimation, but may reduce CO₂ entry, photosynthesis, growth, and carbon gain under prolonged salinity. Dashed elements indicate mechanisms inferred from model plants or requiring further wheat-specific validation.

Abscisic acid (ABA) links salinity-induced osmotic stress with whole-plant water regulation in wheat. When external NaCl lowers soil water potential, roots experience an early water-deficit signal before severe Na⁺ toxicity develops. ABA integrates this osmotic signal with Ca^2^⁺, ROS, phosphorylation cascades, ion-channel regulation, hydraulic adjustment, and compatible-solute accumulation (Figure 3).^23^ ^,^ ^26^ In guard cells, the core ABA pathway involves PYR/PYL/RCAR receptor-mediated inhibition of PP2Cs, activation of SnRK2 kinases, ROS production through NADPH oxidases, Ca^2^⁺ influx, and regulation of ion channels that reduce guard-cell turgor and close stomata.^98-100^ In wheat, ABA signaling is supported by studies on *TaPYL* receptors, *TaPP2Cs*, SnRK2 kinases, and physiological responses such as reduced stomatal conductance, improved water-use efficiency, osmolyte accumulation, and antioxidant activation under water-limited or saline conditions.^69^ ^,^ ^101^

### 5.1. ABA as a central regulator of salinity-induced osmotic stress

Salinity-induced osmotic stress promotes ABA accumulation in roots and shoots allowing wheat plants to respond to declining water availability before severe ion toxicity occurs. Root-derived ABA can move to shoots through the transpiration stream, while leaf tissues can also synthesize ABA locally under dehydration.^102^ ^,^ ^103^ This dual origin allows ABA to function as both a local and long-distance signal controlling stomatal closure, osmotic adjustment, antioxidant defense, root water uptake, and growth restraint.

At the molecular level, ABA is perceived by PYR/PYL/RCAR receptors, which inhibit clade A PP2Cs and release SnRK2 kinases from repression. Activated SnRK2s phosphorylate downstream targets, including transcription factors and ion channels, thereby connecting osmotic perception with rapid stomatal responses and longer-term stress-responsive gene expression. Wheat contains stress-responsive SnRK2 family members, and expression analyzes showed that several wheat *SnRK2* genes respond to ABA, drought, salt, and cold, indicating that ABA-dependent kinase signaling is active in wheat stress adaptation.^100^ More recent wheat work also identified *TaPYL5* as an ABA receptor-like protein that interacts with PP2C partners and is associated with drought-responsive ABA signaling,^101^ supporting conservation of the PYR/PYL-PP2C-SnRK2 module in wheat.

### 5.2. ABA-Ca^2^⁺-ROS signaling in guard cells

Guard cells are key sites where ABA, Ca^2^⁺, and ROS interact to regulate water loss. In the canonical guard-cell model, ABA activates NADPH oxidases, leading to apoplastic ROS production. ROS then stimulate Ca^2^⁺ influx through Ca^2^⁺-permeable channels, increasing cytosolic Ca^2^⁺ and activating Ca^2^⁺-dependent protein kinases.^104^ ^,^ ^105^ These events, together with SnRK2 activity, regulate anion channels such as SLAC1, promote anion efflux and membrane depolarization, drive K⁺ efflux, and reduce guard-cell turgor, causing stomatal closure (Figure 3).^106^ ^,^ ^107^

Most detailed ABA-ROS-Ca^2^⁺ guard-cell mechanisms have been established in *Arabidopsis* and other model systems rather than directly in wheat. Therefore, in wheat this pathway should be presented as a conserved mechanistic framework supported by physiological and genetic evidence, not as a fully resolved wheat-specific pathway. Wheat evidence does support its functional relevance: manipulation of ABA receptor signaling in wheat can improve water-use efficiency and overexpression of an ABA receptor increased water-use efficiency in polyploid wheat, showing that ABA perception can be used to tune stomatal water loss in this crop.^108^ Under salinity, this ABA-mediated stomatal response helps conserve water and protect leaf water status, but it also restricts CO₂ diffusion and can reduce photosynthetic carbon gain if prolonged.^69^ ^,^ ^109^

### 5.3. Osmotic adjustment through ABA-regulated metabolites

ABA also supports water balance by promoting compatible-solute accumulation. Under salinity, metabolites such as proline, soluble sugars, glycine betaine, trehalose, sugar alcohols, and amino acids reduce cellular osmotic potential, maintain turgor, stabilize proteins and membranes, and buffer redox imbalance.^67^ ^,^ ^110^ ABA influences these responses by regulating osmolyte biosynthesis, sugar metabolism, antioxidant defense, and stress-responsive transcription. Thus, osmotic adjustment is a hormone-regulated metabolic process rather than only passive solute accumulation.^111-113^

Proline is a major ABA-associated osmoprotective metabolite. It contributes to osmotic balance, ROS detoxification, membrane stabilization, and stress signaling.^114^ ^,^ ^115^ Soluble sugars function as osmolytes, carbon reserves, signaling molecules, and substrates for energy metabolism, whereas glycine betaine and trehalose can protect photosynthetic structures and enzymes under osmotic stress.^116-118^ Salt-tolerant genotypes often show stronger or better-coordinated accumulation of proline, soluble sugars, antioxidant metabolites, and protective enzymes than sensitive genotypes, indicating that osmotic adjustment and redox buffering are important components of wheat salinity adaptation.^68^ ^,^ ^81^ ^,^ ^119^ Because osmolyte accumulation requires carbon skeletons, nitrogen assimilation, and ATP, ABA-regulated metabolic protection must be coordinated with photosynthesis and respiratory energy supply.^82^ ^,^ ^120^

### 5.4. ABA control of leaf water status and hydraulic responses

ABA-mediated stomatal regulation is closely linked with plant hydraulic status. The hydraulic signals that result from the decrease in water potential of the root tissue when salinity decreases water uptake interact with the biosynthesis and transport of ABA to cause stomatal closure.^121^ This decreases transpiration and water loss which helps to maintain relative water content of leaves. In wheat, such coordination is important because leaf water status supports photosynthetic tissue integrity, delays wilting, and protects reproductive development under stress.^109^ ^,^ ^122^ ^,^ ^123^

ABA also interacts with root hydraulic conductance, aquaporin activity, and root-to-shoot water transport, although the direction and magnitude of these responses depend on stress severity, genotype, developmental stage, and tissue type. Wheat studies show that salinity can alter root hydraulic conductivity, supporting the view that water transport capacity is part of the salinity response rather than a passive consequence of reduced soil water potential.^122^ More recent work on wheat seedlings under single and combined drought and salt stress also emphasizes the importance of root hydraulic conductivity, transpiration efficiency, stomatal conductance, and water-use efficiency in stress adaptation.^123^ Under moderate stress, ABA-mediated stomatal closure can improve water-use efficiency; under severe or prolonged stress, excessive stomatal closure can limit CO₂ assimilation and reduce carbohydrate supply for growth, ion transport, and osmotic adjustment.^1^ ^,^ ^69^

### 5.5. Trade-offs controlled by ABA-Ca^2^⁺-ROS crosstalk

ABA-Ca^2^⁺-ROS crosstalk improves short-term survival under salinity by reducing transpiration, promoting osmotic adjustment, activating antioxidant defenses, and restraining growth. In wheat seedlings under NaCl stress, exogenous ABA reduced transpiration flow, improved Na⁺ homeostasis, and enhanced antioxidant metabolism, supporting a direct role for ABA in salinity acclimation.^69^ However, these protective effects involve trade-offs. Stomatal closure helps to save water but also decreases the diffusion of CO₂, and can lead to reduced photosynthetic carbon fixation under salinity; wheat screening studies relate transpiration rate, relative water content, osmotic potential, NDVI, photosynthetic rate, biomass and yield to salinity responses.^124^ ^,^ ^125^ Growth suppression reduces water and energy demand but may limit tillering, leaf expansion, biomass accumulation, and yield potential; wheat salinity reviews and genotype studies consistently report reductions in seedling growth, photosynthesis, reproductive development, grain yield, and grain quality under salt stress.^1^ ^,^ ^126^ If photosynthesis, stem reserve remobilization, and transport of assimilates to the grain are limited during post-anthesis, then ABA-associated senescence may help with remobilization of nutrients under severe stress, but premature senescence may decrease source capacity and thus affect grain filling.^127-129^

The outcome of ABA-Ca^2^⁺-ROS signaling therefore depends on timing, intensity, tissue specificity, genotype, and developmental stage. ABA signaling is important for acclimation when it occurs at early or moderate levels, but prolonged accumulation of ABA may cause a reduction in productivity due to limitation of photosynthesis and growth. This balance is particularly critical in wheat during reproductive development when the processes of stomatal regulation, source-leaf photosynthesis, assimilate supply and grain filling are required to be maintained simultaneously.^127^ ^,^ ^130^ Overall, ABA uses Ca^2^⁺ and ROS to convert salinity-induced osmotic stress into coordinated stomatal closure, hydraulic adjustment, compatible-solute accumulation, antioxidant protection, and growth restraint. The strongest wheat-specific evidence currently supports ABA-mediated regulation of transpiration, Na⁺ homeostasis, antioxidant metabolism, water-use efficiency, and salt-responsive physiological traits, whereas detailed ABA-ROS-Ca^2^⁺ guard-cell signaling remains largely inferred from model-plant studies and requires direct wheat validation.^69^ ^,^ ^108^ ^,^ ^109^ ^,^ ^124^

## 6. Hormone-metabolite regulation of growth plasticity under salinity

Growth plasticity is a central component of wheat salinity adaptation because salt stress forces plants to redistribute resources between survival and productivity.^131^ Wheat under saline conditions must reduce water loss, Na⁺ toxicity, maintain K balance, protect photosynthesis and preserve meristem activity while reducing growth that cannot be metabolically sustained.^23^ ^,^ ^33^ This adjustment is controlled by interacting hormone and metabolite networks. Hormones provide developmental and stress-response signals, whereas metabolites supply osmotic protection, redox buffering, carbon-nitrogen information, and energy status.^132^ ^,^ ^133^ Thus, hormone-metabolite crosstalk acts as a growth-decision network that regulates growth, growth rate reduction, remodeling of plant architecture, quiescence and senescence of wheat tissues. This integrated view is in line with recent hormone-stress models that demonstrated crosstalk among ABA, auxin, cytokinins, ethylene, gibberellins, brassinosteroids, jasmonates and salicylic acid in regulating abiotic stress responses, instead of individual hormone pathways.^23^ ^,^ ^78^ ^,^ ^132^

### 6.1. Hormonal rebalancing during wheat salinity adaptation

Salinity causes broad hormonal rebalancing in wheat. ABA generally becomes dominant during the early osmotic phase, promoting stomatal closure, osmotic adjustment, antioxidant defense, and growth restraint. Root elongation, lateral root formation and root architecture are regulated by auxin, enabling the adjustment of root spatial growth under saline conditions. Cytokinins frequently promote cell division, chlorophyll maintenance and shoot activity; their decrease under stress can lead to a decrease in shoot growth and to premature senescence.^3^ ^,^ ^78^ ^,^ ^134^ Ethylene participates in root remodeling, stress signaling, and senescence, whereas reduced gibberellin activity and DELLA stabilization restrain cell expansion and help redirect resources toward stress protection. In addition, brassinosteroids, jasmonates, salicylic acid, and strigolactones regulate root growth, redox homeostasis, defense activation, shoot branching and resource allocation.^23^ ^,^ ^132^ ^,^ ^135^

This hormone control of growth under stress has been confirmed in wheat by several studies. The ABA, auxin, cytokinin, ethylene, gibberellin, brassinosteroid, jasmonate, and salicylic acid signaling pathways have all been implicated in stress adaptation and growth regulation.^3^ ^,^ ^51^ ^,^ ^134^ More directly, recent work identified *TaGSK3* as a molecular switch controlling wheat lateral root developmental plasticity under salt stress through brassinosteroid- and auxin-related signaling. Under salt stress, lateral root primordia can be developmentally paused and then rapidly resume growth after stress release, linking hormonal signaling with reversible root-system plasticity. This offers wheat-specific evidence that hormone crosstalk not only controls stress-defense, but also adaptive root architecture.^79^ ^,^ ^136^ ^,^ ^137^

### 6.2. Hormone crosstalk in growth regulation

Hormone crosstalk determines how wheat reallocates growth under salinity. ABA-auxin interactions can affect root elongation, lateral root initiation and root gravitropic responses, which in turn influence root system under salt stress. ABA-cytokinin antagonism is involved in limitation of shoot growth and regulation of senescence, since ABA promotes stress protection while cytokinins promote maintenance of chlorophyll, cell division and source activity.^3^ ^,^ ^23^ ^,^ ^78^ Ethylene-auxin interactions influence root-zone sensitivity and can either inhibit or redirect growth depending on salt intensity and tissue context. Gibberellin suppression and DELLA accumulation cause a decrease in cell expansion and a decrease in energy demand, both of which are beneficial to stress survival, but can also result in a decrease in biomass and yield if growth is suppressed for too long.^78^ ^,^ ^138^ ^,^ ^139^

Brassinosteroid signaling is especially relevant for wheat root growth under salinity. Wheat brassinosteroid-related genes, like *TaDWF4* and *TaBAK1*, can improve root salt tolerance when expressed in *Arabidopsis*, and exogenous epibrassinolide can partially rescue salt-inhibited wheat root elongation by improving ROS homeostasis, suggesting a conserved role for brassinosteroid signaling in redox-linked root protection.^80^ ^,^ ^136^ Together with the *TaGSK3* evidence, these findings indicate that wheat root plasticity under salinity depends on interaction among brassinosteroid, auxin, ROS, and growth-regulatory kinase pathways rather than on a single hormone signal.^79^ ^,^ ^136^ ^,^ ^137^

### 6.3. Metabolites as regulators of growth and stress acclimation

Metabolites are not only stress products; they also act as regulators of growth and acclimation. Proline contributes to osmotic adjustment, ROS detoxification, membrane stabilization, and stress signaling. Soluble sugars are involved in the regulation of gene expression, hormone sensitivity and meristem activity, and can act as carbon skeletons and energy source.^119^ ^,^ ^140^ ^,^ ^141^ GABA links carbon-nitrogen balance, pH regulation, mitochondrial metabolism, and stress signaling. Organic acids are involved in respiration, pH regulation and ion homeostasis, while phenolics and flavonoids are involved in redox buffering and potentially in hormone signaling.^83^ ^,^ ^142^ ^,^ ^143^

Wheat metabolomic studies support the importance of these compounds under salinity. Comparative metabolic analysis revealed that, when wheat is subjected to salt stress, there is a metabolic switch to sugar accumulation and energy-intensive root and leaf responses, while alkali stress has a stronger effect on the repression of photosynthesis, sugar production, nitrogen metabolism, and glycolysis.^133^ ^,^ ^142^ ^,^ ^144^ More recent wheat salt-alkali studies reported that tolerant genotypes maintain higher soluble sugars, proline, and SOD activity with lower malondialdehyde, indicating better osmotic and oxidative stress management than sensitive genotypes. Dynamic wheat metabolome profiling under salt-alkali stress also revealed a remodeling of proline, sugars, antioxidant enzymes, and stress-related metabolites during seedling growth, supporting the role of metabolite remodeling in early adaptation. These findings show that metabolic adjustment in wheat is closely tied to growth maintenance, redox protection, and genotype-dependent tolerance.^68^ ^,^ ^81^ ^,^ ^83^ ^,^ ^144^ ^,^ ^145^

### 6.4. Hormone-metabolite interactions

Hormone and metabolite pathways are tightly interconnected during salinity adaptation. Sugars influence hormone sensitivity through ABA, auxin, cytokinin, gibberellin, SnRK1 and TOR pathways. High sugar availability can support root growth, osmolyte synthesis, membrane repair, and active ion transport, whereas carbon limitation promotes growth arrest, senescence, and resource conservation.^23^ ^,^ ^146-148^ Trehalose-6-phosphate is especially important as a sucrose-status signal, which is associated with availability of carbon, activity of meristems, growth control and source-sink coordination.^149^ ^,^ ^150^

Proline also interacts with ABA and ROS signaling. ABA can promote proline biosynthesis, while proline supports osmotic balance and helps buffer oxidative stress. GABA might link carbon and nitrogen metabolism to pH, mitochondrial respiration and redox status.^84^ ^,^ ^115^ ^,^ ^151^ In wheat, salt stress has been demonstrated to change mitochondrial metabolism such as tricarboxylic acid cycle and GABA shunt, indicating that respiratory flexibility may be involved in stress adaptation and energy balance.^84^ ^,^ ^152^ ^,^ ^153^ Redox-active metabolites such as flavonoids, phenolics, ascorbate, and glutathione can also influence hormone signaling by modulating ROS levels. Thus, metabolites not only support hormone-regulated responses, but they also act as feed-back molecules for hormone sensitivity, redox signaling and growth decisions.^83^ ^,^ ^154^ ^,^ ^155^

### 6.5. Growth plasticity and adaptive architecture

Growth plasticity under salinity is coordinated by changes in root and shoot architecture. Roots may reduce elongation in highly saline zones while maintaining or promoting growth in less stressful regions. This plasticity aids in optimization of water and nutrient uptake, and reduces exposure to toxic levels of ions.^126^ ^,^ ^156^ ^,^ ^157^ Root-to-shoot biomass allocation often shifts toward root maintenance, ion exclusion, osmotic adjustment, and antioxidant defense. Growth of shoots is often limited by reduced tillering, internode elongation, leaf expansion and biomass accumulation. Although these changes reduce productivity potential, they can improve survival by lowering transpiration demand and conserving energy.^68^ ^,^ ^157^ ^,^ ^158^

The adaptive value of growth restraint depends on stress intensity and duration. Under moderate salinity, controlled shoot restriction combined with continued root activity may support acclimation and recovery. Severe or prolonged stress causes excessive growth suppression, which leads to a decrease in canopy development, photosynthesis, stem carbohydrate reserves and grain filling.^131^ ^,^ ^158^ ^,^ ^159^ Wheat-specific evidence from *TaGSK3*-mediated root plasticity suggests that adaptive architecture can involve reversible developmental pausing rather than irreversible growth failure. This is crucial in field conditions, where salinity vary spatially and temporally. Therefore, hormone-metabolite regulation is crucial to balance ABA mediated protection, auxin and brassinosteroid mediated root plasticity, cytokinin supported source activity, gibberellin related growth potential and metabolite based energy status.^79^ ^,^ ^136^ ^,^ ^137^

### 6.6. Senescence, acclimation, and stress memory

Senescence is a double-edged component of salinity adaptation. Under severe stress, senescence can remobilize nutrients and carbohydrates from older leaves to younger tissues or developing grains. But early senescence decreases the photosynthetic source capacity and can affect yield. ABA, ethylene, cytokinins, ROS, and metabolites jointly regulate this process. ABA and ethylene are involved in stress induced senescence while cytokinins are linked to chlorophyll retention, delayed leaf senescence and stay green traits.^160-162^ ROS act both as senescence signals and damage agents, depending on antioxidant capacity and redox buffering. Therefore, the timing and intensity of senescence strongly influence wheat performance under salinity.^68^ ^,^ ^126^ ^,^ ^158^

Repeated or moderate stress exposure may also produce acclimation or stress-memory-like responses. Such responses can involve metabolic priming, antioxidant readiness, osmolyte accumulation, altered hormone sensitivity, transcriptional responsiveness, and chromatin-related regulation. Stress memory is better characterized in model plants than in wheat, so claims for wheat should be cautious. Nevertheless, recent wheat evidence supports its relevance: an intergenerational stress-memory study linked salt resilience with antioxidant responses and genetic variation, indicating that memory-associated redox regulation may contribute to salt tolerance in wheat populations.^163^ ^,^ ^164^ Seed priming and biostimulant studies also show that wheat can be preconditioned through enhanced antioxidant capacity, osmolyte accumulation, and improved ionic balance under salinity,^165^ ^,^ ^166^ although the persistence and molecular basis of these effects require further validation. Overall, hormones and metabolites reshape wheat growth under salinity by integrating stress intensity, carbon and nitrogen status, redox balance, developmental stage, and genetic background into coordinated decisions about root architecture, shoot expansion, tillering, senescence, recovery, and yield formation.^23^ ^,^ ^157^

## 7. Sugar-redox-hormone protection of photosynthesis and grain filling

**Figure 4.**
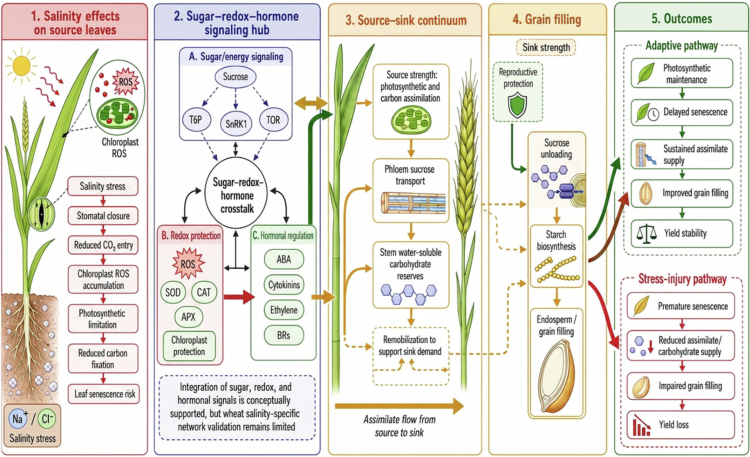
Sugar-redox-hormone regulation of wheat grain filling under salinity. Salinity affects source leaves by inducing stomatal closure, reducing CO₂ entry, promoting chloroplast ROS accumulation, limiting photosynthetic carbon fixation, and increasing senescence risk. Sugar/energy signals, including sucrose, T6P, SnRK1, and TOR, interact with redox protection systems and hormonal regulators such as ABA, cytokinins, ethylene, and brassinosteroids to coordinate source-sink balance. These signals influence photosynthetic maintenance, phloem sucrose transport, stem water-soluble carbohydrate remobilization, sucrose unloading, starch biosynthesis, sink strength, and endosperm grain filling. Effective coordination supports sustained assimilate supply, improved grain filling, and yield stability, whereas stress injury promotes premature senescence, reduced assimilate supply, impaired grain filling, and yield loss. Dashed elements indicate inferred or less directly validated mechanisms in wheat under salinity.

Photosynthesis and grain filling are very sensitive to salinity due to the requirement of coordinated water status, ion balance, chloroplast function, carbon assimilation, assimilate transport, and reproductive sink activity. Salinity, sugar, redox and hormone signals act in coordinated way to protect photosynthetic tissues, to control source-sink relations, and to maintain grain development (Figure 4).^1^ ^,^ ^3^ ^,^ ^126^ Sugars provide metabolic substrates and carbon-status information; redox systems protect chloroplasts and reproductive tissues from oxidative damage; and hormones regulate stomatal conductance, senescence, assimilate partitioning, and grain development.^68^ In wheat, salinity causes a decrease in photosynthetic capacity, affects production and distribution of carbohydrates and may affect grain filling, making source-sink relationships important for yield stability.^1^ ^,^ ^94^ ^,^ ^131^

### 7.1. Salinity effects on photosynthesis and carbon metabolism

Salinity restricts wheat photosynthesis through both stomatal and non-stomatal mechanisms.^167^ ^,^ ^168^ In early osmotic phase, ABA-induced stomatal closure will decrease transpiration and maintain leaf water status, but will also decrease CO₂ entry and reduce carbon fixation. With prolonged stress, Na⁺/Cl^−^ accumulation and oxidative stress impair chlorophyll stability, photosystem activity, thylakoid integrity, Calvin-cycle enzymes, and electron transport. These non-stomatal limitations decrease photosynthetic capacity even when stomatal closure is not the only limitation.^69^ ^,^ ^125^ ^,^ ^167^ Chloroplasts are major ROS-generating sites under salinity because restricted CO₂ fixation can over-reduce the photosynthetic electron transport chain. Increased ROS levels due to excess excitation energy lead to the degradation of pigments, photosystem proteins, thylakoid membranes, and carbon-fixation enzymes when antioxidant capacity is insufficient.^68^ ^,^ ^69^ ^,^ ^167^

Wheat studies show that salinity decreases chlorophyll and carotenoid synthesis and reduces photosynthetic efficiency, while treatments or genotypes that improve antioxidant capacity and ion balance often preserve chlorophyll content and growth under salt stress.^68^ ^,^ ^77^ ^,^ ^169^ ^,^ ^170^ Additionally, salinity causes changes in sucrose synthesis, starch turnover, phloem loading, and assimilate transport, which ultimately leads to a decrease in carbon availability to roots, stems, developing leaves, and grains. In wheat, disruption of carbohydrate synthesis during vegetative growth and impaired carbohydrate translocation at grain filling are major routes through which salt stress reduces yield.^131^ ^,^ ^171^

### 7.2. Sugar signaling under salinity

Sugars are not only products of photosynthesis, but also serve as signals to control wheat's growth, defense and reproductive development in response to salinity. Fructose, glucose, sucrose, and trehalose-6-phosphate provide information about energy status, carbon availability, and source-sink balance. Under salt stress, sugar accumulation can contribute to osmotic adjustment, membrane stabilization, ROS buffering, and energy supply for Na⁺ exclusion, K⁺ retention, vacuolar sequestration, antioxidant defense, and root maintenance.^68^ ^,^ ^126^ ^,^ ^148^ At the same time, sugar signals regulate stress-responsive gene expression, meristem activity, senescence, and grain sink strength.^149^ ^,^ ^172^ ^,^ ^173^

Trehalose-6-phosphate is particularly relevant for wheat because it links sucrose status with growth and grain development. In wheat grains, T6P levels change dramatically during development and correlate with sucrose content, while T6P strongly inhibits SnRK1 activity in vitro, supporting its role as a sugar-status signal during grain filling.^172-174^ This does not prove a salinity-specific T6P mechanism in wheat grain filling, but it provides a strong wheat-based framework for understanding how carbon availability may be translated into grain developmental decisions under stress. Under salinity, sugar signaling through T6P, SnRK1, TOR, and ABA-related pathways may help determine whether assimilates are directed toward growth, osmolyte synthesis, antioxidant protection, ion transport, stem storage, or grain filling.^131^ ^,^ ^148^ ^,^ ^149^

### 7.3. Redox protection of the photosynthetic apparatus

Redox protection is essential for sustaining photosynthesis under salinity. When CO₂ fixation is restricted, excess electrons can be transferred to oxygen, producing superoxide, hydrogen peroxide, and singlet oxygen. Wheat leaves must therefore activate enzymatic antioxidants, including superoxide dismutase, catalase, ascorbate peroxidase, glutathione reductase, peroxidases, and glutathione-S-transferase, as well as non-enzymatic antioxidants such as ascorbate, glutathione, carotenoids, tocopherols, phenolics, and flavonoids.^154^ ^,^ ^175^ ^,^ ^176^ These systems protect chlorophyll, photosystems, membranes, and metabolic enzymes while also shaping ROS-dependent signaling.

Wheat-specific evidence supports the importance of antioxidant protection. Improved nitrogen supply under salinity reduced oxidative effects and was associated with better chlorophyll/carotenoid status, photosynthetic efficiency, osmolyte accumulation, and antioxidant activity in wheat. Silicon supplementation also alleviated wheat salinity stress by reducing Na⁺ uptake, improving K⁺ absorption, enhancing chlorophyll status, increasing antioxidant enzyme activity, and improving growth.^67^ ^,^ ^169^ ^,^ ^177^ Cultivar comparisons further show that wheat genotypes differ in salt-induced growth reduction, antioxidant capacity, phenolic accumulation, and grain-yield response, supporting the view that redox buffering is a genotype-dependent component of salt tolerance.^125^ ^,^ ^140^ ^,^ ^178^ Thus, redox regulation is not only damage control; it is part of the signaling network that protects source leaves and delays premature senescence.

### 7.4. Hormonal regulation of photosynthetic adjustment

Hormones regulate photosynthetic adjustment under salinity by controlling stomatal conductance, chlorophyll retention, antioxidant activity, senescence, and source strength. ABA is the immediate osmotic-stress hormone because it induces stomatal closure and reduces water loss. This protects leaf hydration but can also restrict CO₂ assimilation, creating a trade-off between water conservation and photosynthetic productivity.^1^ ^,^ ^23^ ^,^ ^69^ In general, cytokinin promotes the maintenance of chlorophyll and retards senescence while ethylene can play a role in stress-induced senescence and source decline. Brassinosteroids can enhance the antioxidant capacity and stabilize the photosynthetic machinery, whereas jasmonates and salicylic acid regulate defense-related responses depending on intensity of stress and hormonal balance.^161^ ^,^ ^179^ ^,^ ^180^

In wheat, protection of photosynthesis by means of hormones is supported by mitigation studies. Brassinosteroid-related treatments and signaling components have been associated with improved ROS homeostasis and root salt tolerance, suggesting that brassinosteroid pathways can support redox-linked stress protection.^8^ ^,^ ^136^ Silicon and biostimulant-based studies also indicate that salt stress-induced antioxidant status, chlorophyll retention, ion balance and photosynthetic performance can be translated to better growth.^3^ ^,^ ^67^ ^,^ ^177^ These findings support the idea that wheat photosynthetic maintenance under salinity depends on coordination among ABA-mediated water conservation, cytokinin/stay-green effects, ethylene-associated senescence, brassinosteroid-supported redox protection, and sugar availability.^69^ ^,^ ^131^ ^,^ ^148^

### 7.5. Source-sink reprogramming under salt stress

Salinity alters source-sink relations by reducing carbon production while increasing carbon demand for stress defense. Stomatal closure, chloroplast damage, decreased leaf area or early senescence reduces assimilation by source leaves. Meanwhile, roots need more ATP and reducing power for the exclusion of Na^+^, retention of K^+^, vacuolar sequestration, osmotic adjustment, membrane repair and antioxidant defense. Grains are also competing for assimilates during reproductive growth. Therefore, carbon allocation among roots, leaves, stems, and grains becomes a key determinant of wheat performance under salinity.^1^ ^,^ ^131^ ^,^ ^158^

Wheat can partially buffer grain filling by remobilizing pre-anthesis carbohydrate reserves, especially stem water-soluble carbohydrates, when current photosynthesis is constrained. Source-sink reviews in wheat emphasize that grain yield depends on the balance between current photosynthesis, stored carbohydrate remobilization, nitrogen remobilization, and sink strength during grain filling, particularly under adverse environments.^130^ ^,^ ^181^ Salinity can weaken both source and sink functions by reducing photosynthesis, disrupting carbohydrate translocation, impairing flowering and fertilization, and limiting starch accumulation in developing grains. Sugar transporters, sucrose cleavage enzymes, starch biosynthetic enzymes, and phloem loading therefore become important points where sugar, hormone, and redox signals converge (Figure 4).^131^ ^,^ ^171^ Evidence from wheat grain-filling studies under water-deficit conditions also shows that ABA can regulate sucrose-to-starch conversion enzymes such as sucrose synthase, soluble starch synthase, and starch branching enzyme, suggesting a plausible mechanism by which stress hormones influence grain sink activity.^182^

### 7.6. Grain filling and yield stability

Grain filling is one of the most salt sensitive stages in wheat, as it requires continued source activity, transport of assimilates, sink strength, starch synthesis and viability of reproductive tissues.^183^ Salinity can impair pollen viability, fertilization, grain number, endosperm development, starch accumulation, and final grain weight through combined osmotic stress, ion toxicity, oxidative damage, hormonal imbalance, and reduced assimilate supply. Hence, salinity tolerance at the reproductive stage needs protection of both source leaves and sink tissues.^184^ ^,^ ^185^

The ability of wheat to sustain post-anthesis photosynthesis, mobilize stem reserves, transport sugars efficiently, and prevent oxidative and ionic stress to developing grains are important for yield stability.^130^ ^,^ ^131^ T6P-SnRK1 signaling provides a wheat-based sugar-status framework for grain development, while ABA and other hormones regulate senescence timing, assimilate partitioning, and grain sink activity.^186^ ^,^ ^187^ However, direct wheat evidence connecting salinity, T6P/SnRK1, ABA, redox signaling, and grain filling remains limited. This should be presented as an important future research direction rather than a fully resolved mechanism. Overall, sugar-redox-hormone signaling protects wheat productivity under salinity by integrating carbon availability, chloroplast redox status, stomatal regulation, senescence control, carbohydrate remobilization, and reproductive sink strength into coordinated maintenance of photosynthesis and grain filling.

## 8. Root-shoot and developmental coordination under salinity

**Figure 5.**
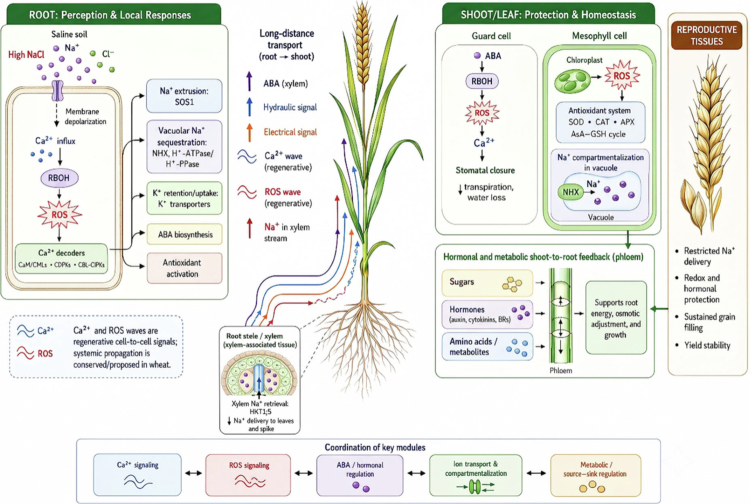
Tissue-specific signaling and long-distance coordination under salinity stress in wheat. Root exposure to NaCl triggers membrane depolarization, Ca^2^⁺ influx, RBOH-dependent ROS production, ABA biosynthesis, antioxidant activation, Na⁺ extrusion through SOS1, vacuolar sequestration through NHX, and K⁺ retention. Root-derived ABA, hydraulic, electrical, Ca^2^⁺, and ROS signals coordinate shoot responses, while HKT1;5-mediated xylem Na⁺ retrieval restricts Na⁺ delivery to leaves and reproductive tissues. In leaves, ABA-ROS-Ca^2^⁺ crosstalk regulates stomatal closure, whereas antioxidant systems and vacuolar compartmentalization protect mesophyll cells. Hormonal and metabolic feedback through the phloem supports root activity, osmotic adjustment, and ion transport, contributing to reproductive protection, grain filling, and yield stability. Solid arrows indicate strongly supported wheat mechanisms, whereas dashed arrows denote inferred or model-based relationships.

Salinity adaptation in wheat requires coordination across roots, shoots, vascular tissues, reproductive organs, and developmental stages. Although salt is first perceived by roots, its effects extend to leaf water status, photosynthesis, ion distribution, senescence, flowering, grain set, and grain filling. Successful adaptation therefore depends on long-distance root-shoot communication, shoot-to-root carbon feedback, tissue-specific signaling, and developmental-stage-specific responses.^157^ ^,^ ^188^ Hydraulic, hormonal, electrical, Ca^2^⁺, ROS, and metabolic signals together regulate stomatal behavior, ion transport, antioxidant defense, growth adjustment, source-sink balance, and reproductive protection (Figure 5).^23^ ^,^ ^189^ In wheat, this whole-plant integration is supported by genetic evidence for root/vascular Na⁺ control, transcriptomic differences between roots and leaves, and physiological evidence that salinity affects germination, vegetative growth, reproductive development, grain yield, and grain quality.^14^ ^,^ ^35^ ^,^ ^131^ ^,^ ^190^ ^,^ ^191^

### 8.1. Root-to-shoot signaling and long-distance Na⁺ regulation

Following local salt perception, wheat roots must rapidly communicate changes in water availability and ion status to vascular and aerial tissues. This communication occurs through interacting hydraulic, hormonal, electrical, Ca^2^⁺, ROS, and metabolic signals. Reduced root water uptake generates hydraulic signals, while ABA synthesized or accumulated in roots can move through the xylem and promote stomatal closure, osmotic adjustment, and antioxidant activation in leaves. Membrane depolarization contributes to electrical signaling, whereas sugars, amino acids, organic acids, and peptides provide additional information about metabolic and nutritional status.^192-194^ Ca^2^⁺ and ROS may also transmit stress information through regenerative cell-to-cell waves involving repeated Ca^2^⁺ entry, intracellular Ca^2^⁺ release, and RBOH-dependent ROS production. These waves should not be interpreted as the continuous physical transport of the same Ca^2^⁺ ions or ROS molecules from roots to shoots, and their real-time propagation remains better established in model plants than in wheat.

Rapid systemic signaling must be distinguished from the slower physical movement of Na⁺. Hydraulic, electrical, hormonal, and regenerative Ca^2^⁺-ROS signals may initiate leaf acclimation before substantial Na⁺ accumulation occurs, allowing shoots to reduce transpiration and water loss, activate antioxidant defenses, and adjust osmotic status.^69^ ^,^ ^126^ ^,^ ^195^ By contrast, Na⁺ enters the xylem transpiration stream and is transported toward leaves and reproductive tissues. Its long-distance distribution is therefore determined by root exclusion, xylem loading and retrieval, and intracellular compartmentalization. SOS1-associated Na⁺ extrusion restricts cytosolic and xylem Na⁺ entry, whereas NHX-type antiporters sequester Na⁺ in vacuoles. Together, these mechanisms complement vascular Na⁺ retrieval and protect metabolically active tissues.

HKT1;5-mediated xylem Na⁺ retrieval provides the strongest wheat-specific example of this regulation. The *Nax2* locus in durum wheat and the *Kna1* locus in bread wheat are associated with HKT1;5-type transporters expressed around xylem tissues, where they retrieve Na⁺ from the transpiration stream and restrict its delivery to shoots.^35^ ^,^ ^190^ Functional and field evidence showed that *TmHKT1;5-A* reduced leaf Na⁺ accumulation and improved durum wheat grain yield under saline conditions, demonstrating that vascular ion regulation can translate into whole-plant productivity.^27^ ^,^ ^190^ More recently, the TaSPL6-D-TaHKT1;5-D regulatory module was identified as a determinant of salinity tolerance and yield-related performance in bread wheat.^30^ ^,^ ^196^ Thus, root-to-shoot coordination integrates rapid stress signaling with the physical control and compartmentalization of Na⁺, thereby protecting leaf water relations, photosynthesis, reproductive tissues, and grain formation under salinity.

### 8.2. Shoot-to-root feedback

Shoot-to-root feedback is equally important because root salt tolerance depends on carbon and energy supplied by photosynthetic tissues. Under salinity, roots require ATP, reducing power, and carbon skeletons to sustain Na⁺ exclusion, K⁺ uptake, vacuolar sequestration, osmotic adjustment, membrane repair, and antioxidant defense. Stomatal closure, chloroplast dysfunction or early senescence may decrease carbon assimilation, which may lead to less photosynthate delivery to roots, and consequently to a reduction in ion transport and root growth maintenance. Thus, root Na⁺ exclusion is not only a root-local trait but also depends on shoot photosynthetic capacity and source strength.^34^ ^,^ ^157^

Shoots also regulate roots through hormonal and metabolic feedback. Leaf-derived sugars, auxin, cytokinins, ABA, and other mobile signals can influence root elongation, lateral root development, transporter expression, and root hydraulic conductance.^197^ ^,^ ^198^ The hydraulic regulation of water transport in wheat roots is an active component of stress adaptation and is altered by salinity, as shown in hydraulic studies of wheat roots.^122^ ^,^ ^123^ Studies of wheat seedlings under single and combined drought/salinity stress further emphasize the importance of root hydraulic conductivity, transpiration efficiency, stomatal conductance, and water-use efficiency in coordinating root and shoot responses. Together, this evidence indicates that wheat salinity tolerance depends on bidirectional communication: roots regulate shoot water and ion status, whereas shoots provide carbon, hormones, amino acids, and metabolic signals that sustain root energy supply, ion transport, osmotic adjustment, and growth.^123^ ^,^ ^126^ ^,^ ^199^

### 8.3. Tissue-specific signaling networks

Salinity signaling is highly tissue-specific. Root epidermal and cortical cells are directly exposed to saline solution and are responsible for early osmotic perception, Na⁺ entry control, Ca^2^⁺ signaling, ROS production, and root growth adjustment. Xylem parenchyma cells control Na⁺ loading and retrieval from the transpiration stream, thus regulating the amount of Na+ that reaches the leaves. Vascular tissues also act as transport and signaling conduits for water, ions, ABA, hydraulic and electrical signals, regenerative Ca^2^⁺-ROS waves, sugars, amino acids, and other mobile metabolites.^27^ ^,^ ^35^ ^,^ ^190^ ^,^ ^195^

Wheat transcriptome studies support this tissue specificity. Comparative analyzes of root and shoot tissues under salt stress revealed thousands of genes that were differentially expressed between tissues, with more DEGs in roots, and enhanced partitioning of Na⁺ into roots.^200^ ^,^ ^201^ These studies demonstrate that wheat salinity responses are tissue- and genotype-dependent rather than uniform across the plant. In leaves, guard cells integrate ABA, ROS, Ca^2^⁺, hydraulic status, and ion fluxes to regulate stomatal closure and water loss, whereas mesophyll cells protect chloroplasts, maintain redox balance, compartmentalize Na⁺, and sustain carbon fixation. Reproductive tissues require restricted Na⁺ delivery, redox and hormonal protection, and continued assimilate supply to preserve grain filling and yield stability (Figure 5).^131^ ^,^ ^168^ Wheat hexaploidy further adds complexity because homoeologs exhibit subgenome-biased expression, silencing under stress, dosage differences, tissue specificity, and functional diversification contributing to salt tolerance.^63^ ^,^ ^202^

### 8.4. Developmental-stage-specific responses

Wheat sensitivity to salinity varies strongly with developmental stage. Salinity during germination causes a decrease in water uptake, delays enzyme activation, interferes with reserve mobilization, and slows radicle emergence. Genetic mapping of wheat germination and seedling traits under salt stress identified multiple QTLs, supporting the view that early-stage salt tolerance has a genetic basis and may differ from later-stage tolerance.^203^ ^,^ ^204^ Root elongation, leaf expansion, ion balance, osmotic adjustment and antioxidant defense are all important for survival at seedling stage. During vegetative growth, wheat may partially acclimate by modifying root architecture, reducing shoot expansion, regulating stomata, enhancing antioxidant capacity, and maintaining Na⁺/K⁺ balance.^68^ ^,^ ^157^

The reproductive stage is often highly vulnerable because flowering, pollen development, fertilization, grain set, and grain filling depend on water status, ion balance, redox protection, and assimilate supply. Salinity stress during reproductive development can affect pollen viability, fertilization, senescence, endosperm development, and decrease in number and weight of grain.^1^ ^,^ ^184^ ^,^ ^185^ The two-phase model helps explain why early osmotic effects rapidly restrict growth, whereas later ion accumulation damages photosynthetic and reproductive tissues. In wheat, developmental-stage-specific signaling is therefore crucial because seedling tolerance does not always predict reproductive-stage tolerance or field-level yield stability.^94^ ^,^ ^158^ ^,^ ^205^

### 8.5. Whole-plant adaptive responses

Whole-plant adaptation to salinity is a consequence of coordinated responses of roots, shoots and reproductive organs. Early root perception triggers systemic signals that regulate stomatal closure, leaf rolling, root remodeling, shoot growth restriction, antioxidant defense, osmotic adjustment, Na⁺ exclusion, K⁺ retention, and vacuolar sequestration. Root remodeling helps in the acquisition of water and nutrients while stomatal closure and leaf rolling helps to reduce water loss. Shoot growth restriction decreases transpiration demand and energy expenditure, while ion exclusion and compartmentalization decrease toxicity of Na⁺ in the cytosol.^31^ ^,^ ^158^ ^,^ ^206^

These adaptive responses involve trade-offs. The stomatal closure is effective in maintaining water status, but it reduces CO₂ assimilation. The reduction of shoot growth increases survival but may negatively affect tillering, leaf area, stem carbohydrate storage and yield potential. Senescence can remobilize nutrients and carbohydrates but reduces source capacity if it occurs too early. Grain filling depends on current photosynthesis, stem carbohydrate remobilization, sink strength, and protection of reproductive tissues. Therefore, salinity tolerance in wheat should be considered a whole-plant trait, not just a single trait of root, leaf or transporters.^94^ ^,^ ^130^ ^,^ ^207^ The strongest wheat-specific evidence currently links tolerance with root/vascular Na⁺ control, K⁺ retention, tissue-specific transcriptional responses, hydraulic regulation, antioxidant capacity, and developmental-stage resilience.^10^ ^,^ ^14^ ^,^ ^68^ ^,^ ^122^ This integrated framework connects early root perception with photosynthetic maintenance, reproductive protection, and yield stability under saline environments.

## 9. Translational perspectives and future directions

To translate the knowledge of salinity signaling networks into wheat improvement, needs moving beyond single gene/single trait approaches. Salt tolerance in wheat is a complex trait controlled by ion homeostasis, osmotic adjustment, redox stability, hormonal regulation, photosynthetic maintenance, root-shoot coordination, and reproductive resilience. Therefore, future improvement should focus on signaling hubs, network interactions, tissue-specific regulation, and field-relevant physiological performance. The most promising targets are not only transporters or stress responsive genes, but also regulatory nodes, which link Ca^2^⁺, ROS, ABA, ion transport, metabolism, growth and yield formation.

### 9.1. Signaling hubs as targets for salt-tolerant wheat improvement

Signaling hubs provide attractive targets for improving wheat salinity tolerance because they control multiple downstream responses. CBL-CIPK modules, SOS-like pathway components, HKT transporters, NHX antiporters, RBOHs, CDPKs, MAPKs, SnRK2s, ABA signaling regulators, redox enzymes, sugar regulators, and metabolic sensors all participate in salt adaptation. These hubs connect early stress perception with Na⁺ exclusion, K⁺ retention, vacuolar sequestration, antioxidant activation, osmotic adjustment, stomatal regulation, and growth control. Transcription factors like NAC, WRKY, DREB, bZIP, MYB and bHLH families are also important since they function as hubs for the convergence of signaling pathways with broad transcriptional reprogramming.

However, hub selection must be cautious. Targeting a major regulator can improve stress tolerance but may also cause growth penalties, altered development, or reduced yield under non-saline conditions. This has been further illustrated in recent wheat studies where the *TaSPL6-D-TaHKT1;5-D* module could be regulated to impact salinity tolerance and yield in saline soils, supporting the importance of upstream regulatory hubs associated with ion transport.^30^ At the same time, recent discussions on wheat salt-tolerance breeding caution that focusing only on classic Na⁺ exclusion targets such as *SOS1* or *HKT1* may be insufficient across diverse saline environments.^94^ Future strategies should thus focus on network hubs which facilitate stress adaptation with maintaining photosynthesis, reproductive development and grain yield.

### 9.2. Multi-omics identification of salinity crosstalk networks

Multi-omics approaches are crucial to uncover regulatory networks that underlie wheat salinity tolerance. Transcriptomics can identify salt responsive genes and transcription factors, proteomics can identify changes in protein abundance, phosphoproteomics can identify target of kinases, metabolomics can map osmolytes and metabolites related to redox, and ionomics can quantify the dynamics of Na^+^, K^+^, Ca^2+^ and other nutrients. Epigenomics, phenomics, spatial omics, and single-cell approaches add further resolution by linking molecular regulation with tissue identity, developmental stage, and physiological performance.

The main value of multi-omics is integration. Integrated datasets can identify genes or metabolites that are responsive to salt stress, and can help to uncover how the Ca^2^⁺ signaling, ROS metabolism, ABA response, ion transport, sugar status, and growth regulation interact as a network. Integrated transcriptomic and metabolomic analyzes in wheat have already shown that salt-tolerant and salt-sensitive genotypes differ in coordinated gene-metabolite responses during early growth.^208^ More broadly, recent wheat multi-omics studies that integrate transcriptome, proteome, phosphoproteome and acetylproteome layers, indicate the growing feasibility of systems-level analysis in common wheat, despite its complexity.^209^ Future studies should connect omics networks with ion fluxes, real-time signaling dynamics, root-shoot communication, and yield traits under realistic salinity conditions.

### 9.3. Wheat-specific validation challenges

Wheat presents unique validation challenges because of its hexaploid genome, homoeolog redundancy, and functional compensation among A, B, and D subgenomes. A gene that is found to be salt responsive can have several homoeologs that express differently, in different tissues, or with varying strengths. Some homoeologs may compensate for one another, whereas others may have diverged in function. This complexity makes it difficult to infer function from expression data alone. Hence, there is a need for homoeolog-specific expression analysis, functional validation and physiological phenotyping of wheat salinity research.

The gap between results in a controlled environment and field performance is another major problem. Many salinity studies are conducted at seedling stages, in hydroponics, or under short-term stress, whereas field salinity is spatially heterogeneous, seasonally dynamic, and often combined with drought, heat, nutrient limitations, or soil structural constraints. The genotype-specific responses can also vary with developmental stage; thus, seedling tolerance does not necessarily equate with reproductive-stage tolerance and yield stability. Recent reviews emphasize that wheat salt-tolerance improvement requires better phenotyping platforms, realistic saline-field validation, and broader use of diverse germplasm and wild relatives.^94^ Therefore, before the candidate signaling genes can be considered as reliable breeding targets, they need to be validated in different tissues, different stages, different environments and yield components.

### 9.4. Genome editing and molecular breeding

Genome editing offers powerful tools for validating salinity signaling hubs and developing improved wheat germplasm. CRISPR/Cas systems can be used to test CBL-CIPK interactions, SOS-like pathway components, HKT and NHX transporters, ABA signaling regulators, transcription factors, redox enzymes, and metabolic regulators. In wheat, editing can target one, two, or all three homoeologs, allowing researchers to dissect dosage effects and functional redundancy. The latter (homeoeolog-specific editing) is especially relevant as the complete knockout of all copies may result in developmental penalities while partial or regulatory editing can result in more balanced stress tolerance.

Genome editing should be combined with marker-assisted selection, genomic selection and physiological screening to be used for breeding. Marker-assisted selection is still relevant for major ion-homeostasis loci, but genomic selection can be relevant to capture complex traits, including water-use efficiency, redox stability, root architecture, source-sink balance and yield in salinity. Although the wheat improvement using CRISPR has progressed at a rapid pace, there are still several challenges, such as transformation efficiency, genotype dependency, regulatory issues, off-target effects, and field validation.^210^ Future molecular breeding should combine Na⁺ exclusion, K⁺ retention, antioxidant stability, ABA responsiveness, photosynthetic maintenance, and reproductive resilience rather than selecting for a single salt-tolerance mechanism.

### 9.5. Priming and agronomic manipulation of signaling networks

Practical approaches to alter salinity signaling networks include use of priming and agronomic treatments. Seed priming can improve germination, seedling establishment, antioxidant readiness, osmolyte accumulation, membrane stability, and stress-responsive gene expression under salinity. Ca^2+^ based priming can improve membrane stability, Ca^2+^ signaling and ion selectivity. Hormonal priming using ABA, salicylic acid, jasmonates, brassinosteroids, or related compounds can precondition stress responses. Redox and metabolic priming can enhance antioxidant capacity, proline accumulation, sugar adjustment and stress memory like responses.

Other treatments, including silicon, melatonin, nitric oxide donors, beneficial microbes, and microbial or natural-product biostimulants, can also influence salt tolerance by modifying redox balance, ion transport, root architecture, hormone signaling, and rhizosphere interactions. A recent review on seed priming under salinity highlights the importance of priming in reprogramming of antioxidant, molecular and metabolic responses to enhance salt-stress performance.^211^ Exogenous melatonin has also been found to enhance seed germination under salt stress, as revealed in wheat-specific studies, which involve mitigating oxidative damage and promoting antioxidant responses.^212^ However, field application requires optimization of dose, timing, genotype specificity, soil conditions, and compatibility with agronomic practices.

## 10. Conclusions

Wheat salinity tolerance is governed by coordinated signaling, transport, metabolic, hormonal, and developmental networks rather than by a single protective mechanism. At the root-soil interface, reduced external water potential, Na⁺/Cl^−^ exposure, membrane depolarization, Ca^2^⁺ influx, ROS production, pH changes, nitric oxide, electrical signals, and phosphorylation events initiate early stress responses. These signals are decoded by Ca^2^⁺-binding proteins, CDPKs, CBL-CIPK modules, MAPKs, SnRKs, redox regulators, and transcription factors, which regulate Na⁺/K⁺ homeostasis, antioxidant defense, ABA signaling, osmotic adjustment, growth plasticity, and systemic acclimation.

The strongest wheat-specific evidence supports *HKT1;5*/*Nax*/*Kna1*-associated Na⁺ retrieval, SOS-like Na⁺ extrusion, root K⁺ retention, antioxidant activation, ABA-associated water-use regulation, compatible-solute accumulation, and genotype-dependent transcriptional responses. By contrast, primary salinity sensors, tissue-specific Ca^2^⁺ signatures, real-time Ca^2^⁺-ROS feedback, systemic Ca^2^⁺/ROS waves, detailed guard-cell ABA-ROS-Ca^2^⁺ signaling, and sugar-redox-hormone control of grain filling remain incompletely validated in wheat and should be treated as conserved but testable models.

A signaling-network perspective is essential because salinity adaptation involves trade-offs. ABA-mediated stomatal closure conserves water but may limit CO₂ assimilation; ROS signaling supports acclimation but can cause oxidative injury; Na⁺ exclusion protects leaves but requires energy and carbon supply; and growth restraint improves short-term survival but may reduce source capacity and yield. Future research should therefore prioritize wheat-specific validation of signaling hubs using live-cell Ca^2^⁺/ROS imaging, ion-flux analysis, multi-omics, homoeolog-specific functional studies, genome editing, and field-based phenotyping across developmental stages.

Translationally, the most promising targets are regulatory nodes connecting early perception with whole-plant performance, including Ca^2^⁺ decoders, CBL-CIPK/SOS-like modules, HKT and NHX transport systems, RBOH-antioxidant networks, ABA signaling components, sugar/energy sensors, hormone-metabolite modules, and stress-responsive transcription factors. Integrating these targets with breeding, genome editing, seed priming, biostimulants, and improved agronomic management may help move wheat salinity research beyond isolated tolerance traits toward coordinated improvement of acclimation, reproductive protection, and yield stability.

## Data Availability

No new data was generated for this study.
